
JOURNAL INFORMATION
==============================
NLM Title Abbreviation: ScientificWorldJournal
Journal ID: ScientificWorldJournal
NLM Journal ID: 101131163
Journal ID: TSWJ

The Scientific World Journal

ISSN: 2356-6140
EISSN: 1537-744X
Publisher: Wiley

ARTICLE INFORMATION
==============================
PMCID: PMC6084140
PMID: 30147537
DOI: 10.1100/tsw.2001.104
Article ID: 515043
Article version: 1
Subjects: Short Report

Involvement of REL/NF-B Transcription Factors in Cellular Senescence

Bernard David 1
Monté Didier 2
Vandenbunder Bernard 1
Abbadie Corinne 1 *
1 CNRS EP560/Institut Pasteur de Lille/Université Lille 2, Institut de Biologie de Lille, 1 rue du Pr. Calmette, BP447, 59021 Lille cedex, France
2 UMR8526/Institut Pasteur de Lille/Université Lille 2, Institut de Biologie de Lille, 1 rue du Pr. Calmette, BP447, 59021 Lille cedex, France
*Corinne Abbadie: corinne.abbadie@ibl.fr
Publication date: 2001
Electronic publication date: 2001 Dec 1
Volume: 1
First page: 67
Last page: 67
Copyright: Copyright © 2001 David Bernard et al.
Copyright year: 2001
License: This is an open access article distributed under the Creative Commons Attribution License, which permits unrestricted use, distribution, and reproduction in any medium, provided the original work is properly cited.
License URL: https://creativecommons.org/licenses/by/3.0/

Funding: Centre national de la recherche scientifique

==============================
Miami *Nature Biotechnology* Short Reports
*TheScientificWorld* (2001) 1(S3), 67SR
ISSN 1532–2246; DOI 10.1100/TSW.2001.104

### INVOLVEMENT OF REL/NF-B TRANSCRIPTION FACTORS IN CELLULAR SENESCENCE

David Bernard[1], Didier Monté[2], Bernard Vandenbunder[1], and Corinne Abbadie[1]*
[1]CNRS EP560, [2]UMR8526/Institut Pasteur de Lille/Université Lille 2, Institut de Biologie de Lille, 1 rue du Pr. Calmette, BP447, 59021 Lille cedex, France*corinne.abbadie@ibl.fr

**INTRODUCTION.** Numerous data have demonstrated that Rel/NF-B transcription factors may protect cells from apoptosis induced by diverse stimuli (1). We have shown that in addition to protecting HeLa cells from TNF-induced apoptosis, c-Rel concomitantly blocks their proliferation and increases their basal rate of apoptosis. Proliferation blockage and apoptosis resistance are both due, at least in part, to the induction of the mitochondrial enzyme MnSOD which acts by decreasing the concentration of $O_2^-$ and increasing that of $H_2O_2$ (submitted paper). Since proliferation blockage, apoptosis resistance and accumulation of oxygen reactive species are distinctive feature of senescent cells, we investigated whether c-Rel could induce premature replicative senescence of primary cells and whether Rel/NF-B factors are involved in the occurrence of normal senescence.

**METHOD.** Proliferation rates were measured by BrdU incorporation assays. Apoptosis was induced by 18 hr of TNF (10 ng/ml) plus cycloheximide (10 g/ml). Apoptotic cells were identified by morphological criteria. Senescence Associated--Galactosidase assays were as described elsewhere (2). Gel shifts were done using nuclear extracts on a B consensus probe (Promega).

**RESULTS.** Normal human epidermal keratinocytes (Clonetics) were infected by an adenoviral vector encoding c-Rel, or GFP as control. Resulting morphologies and effects on proliferation and apoptosis were compared to properties acquired during normal replicative senescence. c-Rel overexpression in young keratinocytes induces morphological changes typical of senescent cells, i.e. enlargement and apparition of polynucleated cells. It also increases the basal rate of apoptosis, decreases the rate of proliferation and induces a resistance against TNF-induced apoptosis, all properties shared by senescent cells as well. Finally, c-Rel overexpression increases Senescent Associated--Galactosidase activity, a biomarker of replicative senescence (2).The physiological relevance of c-Rel involvement in replicative senescence was investigated by searching for any difference in Rel/NF-B activity between young and senescent keratinocytes. We demonstrate that during normal senescence there is (i) an increase in Rel/NF-B DNA binding activity, (ii) an increase in the expression of IB, a target gene of NF-B, and (iii) an increase in MnSOD expression. IB and MnSOD were also induced in c-Rel expressing keratinocytes.

**DISCUSSION.** These results suggest for the first time that Rel/NF-B transcription factors may be involved in the occurrence of replicative senescence. In c-Rel-induced premature senescent cells as well as in normal senescent cells, MnSOD is induced. This could disturb the equilibrium between anti-oxidant enzymes and generate oxidative injury which can induce senescence (3, 4).

**ACKNOWLEDGEMENT.** This work was supported by CNRS, Université Lille 2, ARC, Institut Pasteur de Lille, Conseil Régional Nord/Pas-de-Calais and the European Regional Fund. C.A. is Maître de Conférences at Université Lille 1.

**REFERENCES.**

1. Barkett, M. and Gilmore, T.D. (1999) Oncogene 18, 6910-6924
2. Dimri, G.P., Lee, X., Basile, G., Acosta, M., Scott, G., Roskelley, C., Medrano, E.E., Linskens, M., Rubelj, I., Pereira-Smith, O., et al. (1995) Proc. Natl. Acad. Sci. USA 92, 9363-9367
3. De Haan, J.B., Cristiano, F., Iannello, R., Bladier, C., Kelner, M.J., and Kola, I. (1996) Hum. Mol. Genet. 5, 283-292
4. Chen, Q. and Ames, B.N. (1994) Proc. Natl. Acad. Sci. USA 91, 4130-4134
